# Academic clinical trials: Publication of study results on an international registry—We can do better!

**DOI:** 10.3389/fmed.2022.1069933

**Published:** 2022-11-24

**Authors:** Jean-Marc Hoffmann, Regina Grossmann, Annette Widmann

**Affiliations:** Clinical Trials Center, University of Zurich and University Hospital Zurich, Zurich, Switzerland

**Keywords:** academic clinical trials, IITs, clinical trial results, ClinicalTrials.gov, ICH GCP, Declaration of Helsinki

## Introduction

Clinical research is vital for evaluation and development of new therapeutic medical approaches. Not only industry, but also academia is strongly involved to improve public health and quality of life for individuals.

Independent of the context, each clinical trial is initiated by a sponsor who is overall responsible for generating valid data while ensuring participant's safety. In an academic clinical trial—also called investigator-initiated trial or study (IIT, IIS)—this sponsor role is often assumed by a natural person working at an academic institution, resulting in an individual holding both roles, i.e., being the sponsor and the investigator in one person. In contrast to industry-sponsored studies, seeking for market approval and commercial aspects, IITs focus on non-commercial, patient-centered research questions independent from pharmaceutical industry. Academic research enables further insights regarding efficacy and safety of medical interventions in daily routine and real world settings. Funding for academic research projects is assured through institutional, national, international or private grants/funds. After completion of any clinical study it is essential to share the main results with the scientific community and with general public as well. Results and conclusions from academic clinical trials support the treating physicians to determine evidence-based therapeutic approaches or to avoid unnecessary and expensive therapies. Therefore, omitting unexpected or negative outcomes introduces not only a huge publication bias, but the research community misses a big chance for closing gaps in real-world evidence. In addition, publication and communication of any results consolidates patients' trust in medical science resulting in willingness to participate in research activities.

## Registration status of interventional clinical trials on ClinicalTrials.gov

**Figure 1:**
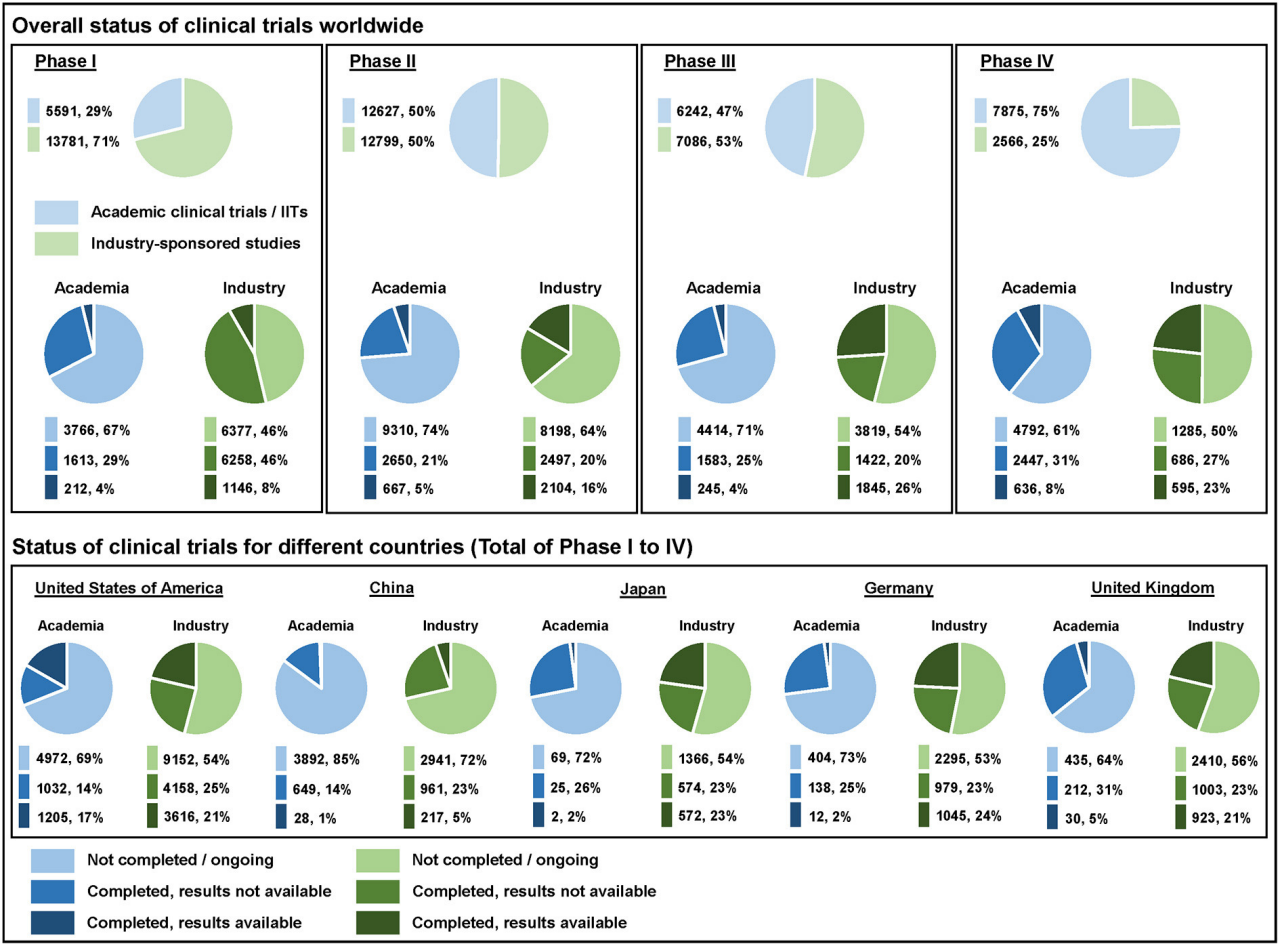
Overall status of clinical trials worldwide as well as their status for different countries listed on ClinicalTrials.gov. The analyzed clinical trials started between January 01, 2015 and December 31, 2021—data were extracted on October 26, 2022.

As one of seven Clinical Trial Units (CTUs) in Switzerland the Clinical Trials Center Zurich supports IITs in all relevant research aspects including registration on an international clinical trials registry. We noticed that many IIT sponsors did not register their studies properly even though this is essential within the scientific community for transparency reasons. To gain a clearer view regarding the registration status of interventional clinical trials we conducted a deeper analysis by extracting data published on ClinicalTrials.gov and analyzed clinical trials worldwide started between January 01, 2015 and December 31, 2021 (Figure 1) (1). ClinicalTrials.gov also allowed us a comparison between IITs and industry-sponsored studies by selecting the type of funding. We analyzed the number of registered clinical trials of all four phases in drug development. With a share of more than 70%, Phase I trials (first-in-human) were mostly industry-sponsored. Exploratory Phase II trials were equally distributed between academia and industry (50% each) and confirmatory Phase III trials almost reached parity as well (IIT: 47%, industry: 53%). Most of post-market Phase IV trials were sponsored by academia with 75%.

We have analyzed the completion status (not completed/ongoing vs. completed with final results not available vs. completed with final results available) of IITs and industry-sponsored studies for direct comparison. More than 45% of the registered clinical trials were ongoing at the time of analysis across all phases. The percentage of ongoing IITs was always higher vs. the share of ongoing industry-sponsored studies—either because the “not completed” trials were actually still ongoing or simply not updated appropriately in the registry. The overall percentage of completed clinical studies with final results available (out of all registered completed trials) was low in both groups, i.e., 18% for IITs and 34% for industry-sponsored studies. Results were published for 12% of IITs in Phase I, 20% in Phase II, 13% in Phase III and 21% in Phase IV. In contrast, industry-sponsors made the results of their studies available for 15% in Phase I, 46% in Phase II, 56% in Phase III and 46% in Phase IV. Academic sponsors reported their clinical trial results significantly less often than industrial sponsors for all four clinical trial phases as shown by the odds ratio (OR)—Phase I: 0.72 (95% CI 0.61–0.84); Phase II: 0.30 (95% CI 0.27–0.33); Phase III: 0.12 (95% CI 0.10–0.14); Phase IV: 0.30 (95% CI 0.26–0.34).

We also compared the completion status for different ICH members. We took the top five countries according to the number of clinical trial registrations on the WHO International Clinical Trials Registry Platform (ICTRP) (2) and compared them on ClinicalTrials.gov. Researchers in the United States of America registered their results for completed IITs most frequently with 54%, followed by research fellows in the United Kingdom (12%), Germany (8%), Japan (7%) and China (4%).

## Discussion

In summary, the sponsors shared their results only for a small percentage of trials flagged as completed, in particular for IITs. Several factors could be the reasons for this non-publication of data. First, the non-publication might be unintentional due to restricted financial and personal capacities, but also due to different priorities in academia, such as the publication of the results in a scientific journal rather than on the primary registries. This trend was also observed by Blumle et al. (3) who analyzed 691 multicenter, randomized controlled trials: results of IITs were mostly published as a journal article while results of industry-sponsored studies were mostly published in study registries. In addition, IITs more often gained impact by inclusion in systematic reviews and guidelines. The rate of unregistered trials and of trials registered retrospectively in medical journals is difficult to assess. A systematic review and meta-analysis by Trinquart et al. (4) analyzed the extent to which published randomized controlled trials (RCTs) were registered and registered prospectively between 2005 and 2017. The pooled proportion of registered RCTs was 53% while the pooled proportion of prospectively registered RCTs was only 20%. Fortunately, the proportion of registered trials significantly increased over time, with a mean increase of 27% between 2005 and 2015. Second, a delay in the publication of clinical trial results could derive from concerns of intellectual property rights for products and therapeutic approaches developed in academia. This practice is comprehensible especially for Phase I studies and maybe also for Phase II and III. However, we also observed a high percentage of non-publication in Phase IV studies: as the pharmaceutical products in this phase already got their market approval, there should be no further incentive in holding back clinical trial data. Third, non-publication of results might be intentional due to detrimental outcomes or premature study discontinuation.

What are the regulatory demands for registration and publication of research studies? The International Council for Harmonisation (ICH) of Technical Requirements for Pharmaceuticals for Human Use regulates the Good Clinical Practice (GCP) of interventional clinical trials with pharmaceuticals. This regulation is based on the Declaration of Helsinki (5). In all ICH regions (EU, USA, and Japan (founding members), Canada and Switzerland (standing members), and many more members) GCP is directly or indirectly implemented in national laws and regulations. According to the Declaration of Helsinki (Section 35 and 36) every research study involving human participants must be registered in an international clinical trial registry including the disclosure of research results (6). In Switzerland sponsors are obliged to register and continuously update their studies both in a national and an international registry (Human Research Act, ClinO Art. 64–67). In the United States, the Food and Drug Administration Amendments Act of 2007 (FDAAA) mandates the registration of clinical trials and the reporting of summary results. DeVito et al. reported that between March 2018 and September 2019 for only 40.9% of clinical trials, which were registered on ClinicalTrials.gov, the results were submitted within 1 year after completion as required by legislation (7). In addition, the International Committee of Medical Journal Editors (ICMJE) requires this registration to be done prior to enrolling the first research participant and agreed upon only publishing results from interventional clinical trials with a trial registration ID. Furthermore, the European Regulation No. 536/2014 requires sponsors to even publish a lay summary in order to inform the general public about study results. Similarly, the European pharmaceutical industry association EFPIA and the US association PhRMA have agreed on joint data transparency guidelines in 2013.

What are the consequences of non-publication in the current situation and what can be done to remedy this shortage? Reporting of clinical study results is indeed a very important scientific and ethical issue. Non-publication of results leads to relevant publication bias. In fact, the valid but missing data cannot be included in systematic reviews or meta-analyses, thus distorting the overall or absolute effect of a medical intervention. In addition, by making the results openly available, other researchers are updated on the current expertise avoiding the repetition of similar clinical studies. Moreover, as IITs are funded by public resources amongst others, the results should be visible to the general public. Non-publication in consequence leads to a loss of trust toward clinical research in general and notably harms the researchers' reputation. Schmucker et al. (8) conducted a systematic review to determine the proportion of studies published as peer-reviewed journal articles. On average, 54% of studies registered in trial registries were published. Similarly, Chen et al. (9) found that 57% of clinical trials registered in ClinicalTrials.gov by leading academic medical centers in the United States shared their results in a journal. Therefore, sponsors of all kind need to increase their efforts toward the publication of their study results. To increase awareness for IITs there should be better support options, not only by continuous advice for sponsor-investigators during the conduct of clinical trials through specialized CTUs, but also by providing good education and training opportunities for researchers and sufficient funding which also covers quality assurance measures. In consequence, quality and regular completion of clinical research studies may improve. In addition, in case of misconduct by the sponsors, warnings should be issued by the competent authorities. The Bennett Institute for Applied Data Science from the University of Oxford developed a software and web interface to assess easily the reporting status of every clinical trial conducted in Europe and registered on the EU Clinical Trials Register (EUCTR) (10). These clinical trial trackers allow users to analyze how conscientious sponsors have reported their results, thus building an incentive for the sponsors to update their records in due time.

## In conclusion

Currently, researchers and academic sponsors do not sufficiently focus on the publication of study results. Thus, measures for improvement of regulatory compliance and quality are needed to increase researchers' awareness and also to enhance the trust of the public and individual study participants in clinical research activities. Finally, the ever-increasing digitalization of our time should facilitate to update online information on the status of clinical studies including their results. We do hope this essay helps to awake consciousness to this relevant issue and can refresh the impetus of sponsors to report their research findings to scientific community and public.

## Author contributions

J-MH and RG conceptualized the essay. J-MH analyzed the data and drafted the manuscript and graphics. RG supervised the planning and conduct of the project and reviewed the manuscript. AW reviewed the manuscript and supervised content and completion of the project. All authors contributed to the article and approved the submitted version.

## Conflict of interest

The authors declare that the research was conducted in the absence of any commercial or financial relationships that could be construed as a potential conflict of interest.

## Publisher's note

All claims expressed in this article are solely those of the authors and do not necessarily represent those of their affiliated organizations, or those of the publisher, the editors and the reviewers. Any product that may be evaluated in this article, or claim that may be made by its manufacturer, is not guaranteed or endorsed by the publisher.
